# P09-06 Getting a grip on sitting behaviour

**DOI:** 10.1093/eurpub/ckac095.136

**Published:** 2022-08-29

**Authors:** Marjolein Duijvestijn, Saskia van den Berg, Wanda Wendel-Vos

**Affiliations:** Centre for Nutrition, Prevention and Health Services, National Institute for Public Health and the Environment, Bilthoven, The Netherlands

**Keywords:** sedentary behaviour, domain specific, sitting

## Abstract

**Background:**

Sedentary time (sitting) has been associated with adverse cardio-metabolic consequences. The general recommendation is to interrupt long periods of sitting. In order to successfully develop interventions and policies to decrease sedentary behaviour, high-risk groups as well as the context of sitting should be identified. The aim of this study was to investigate sedentary behaviour among (subgroups of) the Dutch population and to identify in which domains most sedentary time was spent.

**Methods:**

Data from the 2017 Dutch national Health Interview Survey was used, which includes a nationally representative sample of 8,441 Dutch citizens aged 4 years and older. Sedentary time on an average day was assessed using an adjusted version of the Marshall questionnaire. Sitting domains were defined as: 1) traveling, 2) at work, 3) at school or studying 4) watching television, 5) using a computer/smartphone at home, and 6) otherwise. Total sedentary time was analysed stratified by age, sex and level of education with ANOVA and Bonferroni correction.

**Results:**

On average the Dutch population accumulates 9,0 hours/day of sedentary time. Overall, participants accumulated most sedentary time while watching television (2.2 hours/day) followed by sitting at work and other activities (both 1.7 hour/day). Significant differences (p > 0.001) were found by sex, age group and level of education. Men reported slightly more sedentary hours than women (9.2 vs. 8.8 hours/day). With respect to age groups, adolescents (12-17 years old) reported the highest, whereas children (4-11 years old) reported the lowest sedentary hours (10.1 vs. 7.3 hours/day). Finally, sedentary hours were high for higher educated people (9.7 vs. 8.2 hours/day in lower educated people). Adolescents accumulated most sedentary time at school or during studying (4.0 hours/day), higher educated people accumulated most sedentary time at work (3.4 hours/day).

**Conclusions:**

Our study showed that in general Dutch people spend a lot of time sedentarily, especially adolescents and higher educated people. Most sedentary times was spent while watching television, at school or during studying, and at work. Therefore interventions aiming to decrease sedentary behaviour in the home environment, the occupational as well as the educational setting are of importance to implement.

